# The Recovery of Repeated-Sprint Exercise Is Associated with PCr Resynthesis, while Muscle pH and EMG Amplitude Remain Depressed

**DOI:** 10.1371/journal.pone.0051977

**Published:** 2012-12-17

**Authors:** Alberto Mendez-Villanueva, Johann Edge, Rob Suriano, Peter Hamer, David Bishop

**Affiliations:** 1 Football Performance & Science Department, ASPIRE Academy for Sports Excellence, Doha, Qatar; 2 School of Human Movement and Exercise Science, The University of Western Australia, Perth, Australia; 3 School of Physiotherapy, The University of Notre Dame (Australia), Perth, Australia; 4 Institute of Sport, Exercise and Active Living (ISEAL), School of Sport and Exercise Science, Victoria University, Melbourne, Australia; University of Bath, United Kingdom

## Abstract

The physiological equivalents of power output maintenance and recovery during repeated-sprint exercise (RSE) remain to be fully elucidated. In an attempt to improve our understanding of the determinants of RSE performance we therefore aimed to determine its recovery following exhaustive exercise (which affected intramuscular and neural factors) concomitantly with those of intramuscular concentrations of adenosine triphosphate [ATP], phosphocreatine [PCr] and pH values and electromyography (EMG) activity (a proxy for net motor unit activity) changes. Eight young men performed 10, 6-s all-out sprints on a cycle ergometer, interspersed with 30 s of recovery, followed, after 6 min of passive recovery, by five 6-s sprints, again interspersed by 30 s of passive recovery. Biopsies of the vastus lateralis were obtained at rest, immediately after the first 10 sprints and after 6 min of recovery. EMG activity of the vastus lateralis was obtained from surface electrodes throughout exercise. Total work (TW), [ATP], [PCr], pH and EMG amplitude decreased significantly throughout the first ten sprints (*P*<0.05). After 6 min of recovery, TW during sprint 11 recovered to 86.3±7.7% of sprint 1. ATP and PCr were resynthesized to 92.6±6.0% and 85.3±10.3% of the resting value, respectively, but muscle pH and EMG amplitude remained depressed. PCr resynthesis was correlated with TW done in sprint 11 (r = 0.79, *P*<0.05) and TW done during sprints 11 to 15 (r = 0.67, *P*<0.05). There was a ∼2-fold greater decrease in the TW/EMG ratio in the last five sprints (sprint 11 to 15) than in the first five sprints (sprint 1 to 5) resulting in a disproportionate decrease in mechanical power (i.e., TW) in relation to EMG. Thus, we conclude that the inability to produce power output during repeated sprints is mostly mediated by intramuscular fatigue signals probably related with the control of PCr metabolism.

## Introduction

Repeated-sprint exercise provides an interesting model to investigate the mechanisms governing the decline in power output during whole-body, high-intensity dynamic activities which require high contraction rates similar to those encountered in many athletic activities [1], [2]. The majority of the energy required for all-out sprinting is derived from phosphocreatine (PCr) hydrolysis and anaerobic glycolysis, and repeated sprints therefore result in large changes in both PCr and hydrogen ion (H^+^) concentration [3], [4], [5], [6], [7]. There is also an increased aerobic contribution when sprints are repeated but, nonetheless, even the latter sprints in a repeated-sprint bout remain predominantly anaerobic [8]. Accordingly, most previous explanations of fatigue during repeated-sprint exercise have focused on factors associated with cellular muscle fatigue [9]. These include limitations in anaerobic energy supply from adenosine triphosphate (ATP) and PCr [1], [3], [10], [11], [12], [13] and intramuscular accumulation of selected metabolic by-products including inorganic phosphate (P_i_) and H^+^ [1], [3], [5], [14], [15], [16].

Fatigue induced by repeated-sprint efforts has also been related to neural adjustments, as demonstrated by a reduction in the central nervous system’s drive to the active musculature [17], an impaired muscle activation [18], [19], a reduced EMG amplitude [20], [21], and alterations in the neural strategies for voluntary activation of the contracting muscles [22], [23]. The results from such experiments indicate that, given the high level of activation to sustain maximal power output, a suboptimal motor unit activity could impair the ability to repeatedly generate maximum power outputs [1]. Therefore, while a large component of muscle fatigue during repeated-sprint exercise is likely to be due to local intramuscular factors, neural factors might also contribute to the decline of maximal power output.

While previous studies have measured changes in metabolites [3] and neural factors [17], [21] during RSE, only a limited understanding of the causes of fatigue during repeated-sprint exercise can be obtained by monitoring the many changes which occur concurrently. A limitation of this approach is that it is difficult to discern which of the measured factors, if any, is really responsible for the observed fatigue. One way to study the importance of, and the interplay between, the changes in both muscle metabolism and neuromuscular function on performance during repeated-sprint exercise is to measure the recovery of performance and to relate this to the recovery of both muscle metabolites and neuromuscular function. For example, despite large changes in both PCr and H^+^ concentration during a 30-s all-out test, it has been reported that the recovery of single 10-s sprint performance is not related to changes in muscle pH, but is strongly related to the resynthesis of PCr [13], [14]. While a similar result might be hypothesised for repeated-sprint exercise, we have previously reported that the recovery of single- and repeated-sprint performance follow different time courses [20], suggesting that their recovery might be mediated by different mechanisms.

Consequently, the goal of this study was to investigate for the first time the recovery of repeated-sprint performance, following an exhausting repeated-sprint bout, and to compare this with the recovery of muscle ATP, PCr and H^+^, and neuromuscular activity (via surface electromyography (EMG) signals - a reasonable proxy for net motor unit activity e.g., [24]). We hypothesized that the degree to which these quantities were correlated with mechanical power during repeated-sprint exercise would allow judgements to be made regarding the involvement of putative energy-store, fatigue-metabolite and neuromuscular activity changes in the recovery and therefore composition of mechanical performance during repeated-sprint exercise.

## Methods

### Subjects

Eight healthy, male, recreational, team-sport athletes volunteered to participate in this study. The subjects’ characteristics were as follows (mean±SD): age 19.5±0.9 y, height 183.4±5.4 cm, mass 80.3±8.7 kg, and 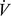
O_2max_ 53.9±5.8 mL·kg^−1^·min^−1^. The exercise protocol, and all possible risks and benefits associated with participation in the study, were explained to each subject. Each subject provided written informed consent prior to participating in the study. Approval for the study’s procedures was granted by the institutional Research Ethics Committee of the University of Western Australia.

### Experimental Design

**Figure 1:**
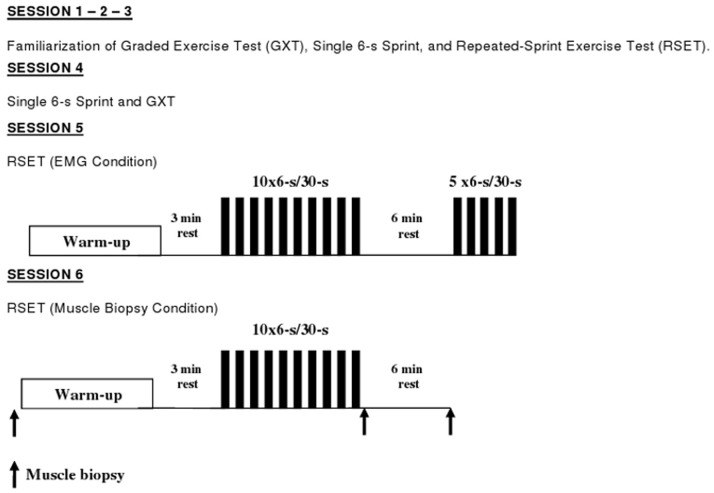
Schematic representation of the experimental design (10×6-s/30-s ten sprints of 6 s in duration each interspersed with 30 s of recovery; 5×6-s/30-s five sprints of 6 s in duration each interspersed with 30 s of recovery). A total of three muscle biopsies was obtained from each subject during the muscle biopsy condition.

All subjects came to the laboratory for three experimental trials, as well as for three familiarization sessions. At least 48 h separated the exercise sessions. An initial laboratory visit was scheduled to obtain data on physical characteristics. Also, during this first session subjects were familiarized with the main repeated-sprint exercise protocol, which was repeated in the second and third sessions. It has previously been established that satisfactory reliability of repeated-sprint cycling protocols is achieved after two familiarization sessions [25]. In the fourth session, subjects performed a single 6-s all-out cycling sprint [26] and after 30 min of passive rest, maximal aerobic power was determined during a graded exercise test (GXT) consisting of graded exercise steps (4-min stages), using an intermittent protocol (1-min passive rest between stages) (data not shown). In the fifth session, subjects performed 10, 6-s all-out sprints on a cycle ergometer, interspersed with 30 s of recovery, followed, after 6 min of passive recovery, by five 6-s sprints, again interspersed by 30 s of passive recovery. In the last trial (sixth) subjects performed only a 10×6-s test of repeated-sprint cycling with muscle biopsies taken at rest, immediately upon cessation of exercise and after 6 min of passive recovery (Fig. 1). Subjects performed their trials at the same time of the day (±2 h), with the laboratory conditions being approximately 20°C and 50% relative humidity during all trials. The subjects were asked to follow their normal diet, to refrain from any form of intense physical activity for the 24 h prior to testing, and to not eat within 3 h before each testing session (this was verified via a 24-h dietary and activity recall).

### Repeated-Sprint Exercise Protocol

The exercise protocol consisted of 10, 6-s sprints on a front-access, air-braked cycle ergometer (Repco, Melbourne, Australia) interspersed with 30 s of recovery. This was followed, after 6 min of passive recovery, by five, 6-s sprints, also interspersed by 30 s of recovery (i.e., 15×6-s protocol). In one further trial (muscle biopsy condition) subjects performed only 10, 6-s sprints with 30 s of recovery (i.e., 10×6-s protocol). Before both tests, subjects performed a standardized warm-up, comprised of 4 min of cycling at a power of 100 to 120 W, followed by three bouts of maximal standing-start accelerations (approx 2 s) and 3 min of rest before performing the main trial. In a separate earlier session, performance in a single 6-s cycling sprint test was recorded and was then used as the criterion score during the main trial. Subjects were instructed to perform an “all-out” effort from the beginning of the test until instructed to stop. During the first sprint, subjects were required to achieve at least 95% of their criterion score, as a check on pacing. All of the subjects satisfied the 95% of the criterion score. Toe clips and heel straps were used to secure the feet to the pedals. Strong verbal encouragement was provided during each trial. All of the sprints were performed from the same initial pedal position with the right crank arm located 45° forward to the vertical axis. During the subsequent 30-s rest period after each sprint, and during the 6-min rest period between sprints 10 and 11, subjects remained quietly seated on the ergometer. Water was provided ad libitum through the trials.

### Mechanical Recordings

The ergometer was interfaced with an IBM-compatible computer system to allow for the collection of data for the calculation of power generated on each flywheel revolution and work performed during each individual sprint repetition (Lab-VIEW, National Instruments Corp., Austin, TX). The power output of the air-braked cycle ergometer is proportional to the cube of the flywheel velocity. Instantaneous work was recorded every 0.2-s during each sprint. Work done was then totalled for each sprint to determine work (TW) and expressed relative to time to determine average power (W). Peak power output was calculated from the highest power output recorded in any 0.2-s measuring epoch. An optical sensor monitored the velocity of the flywheel at a sampling rate of 128 pulses per flywheel revolution. Before testing, the ergometer was dynamically calibrated on a mechanical rig across a range of power outputs (100–2000 W) following the procedures described elsewhere [27]. Peak power output (PPO) and total work (TW) were calculated for each maximal 6-s cycling bout. Repeated-sprint exercise performance before and following the recovery period was estimated from the ratio of TW done during the first five consecutive sprints (i.e., from sprint 1 to 5 and from sprint 11 to 15; TW_5SPT_) to the TW done in the initial sprint (i.e., sprint 1 and sprint 11; TW_1SPT_). Thus, the TW_5SPT_/TW_1SPT_ ratio was used as an index of performance maintenance during repeated sprints.

### Muscle EMG

On the day of the 15×6-s cycle test, the EMG activity from the vastus lateralis (VL) of the right leg was recorded via bipolar Ag-AgCl surface electrodes at an interelectrode distance of 20 mm. We chose the VL muscle because it has been reported that total power output during repeated 6-s cycling sprints separated by 30-s of passive rest (same task as in the present experiment) was significantly correlated with the activation of VL, as evaluated by muscle functional magnetic resonance imaging [19]. Therefore, VL muscle can provide a good indication of whole-thigh neuromuscular activity in the present experimental conditions. Before placing the electrodes, the overlying skin was carefully prepared. The hair was shaved, the skin lightly abraded to remove the outer layer of epidermal cells and thoroughly cleansed with alcohol to reduce the skin-electrode interface impedance to below 2 k-ohms. Electrodes were fixed lengthwise parallel to a line bisecting the proximal and distal tendons over the middle of the muscle belly. The electrodes were taped down with cotton wool swabs to minimise sweat-induced interference. The EMG reference electrode was placed over the right iliac crest. To prevent movement artefact, wires between the electrodes and the computer were secured to the skin with adhesive tape and leads braided to minimise electromagnetic induced interference. The EMG signal was amplified (x 1000) (P511, Grass Instrument Division, West Warwick, RI) and sampled at a rate of 2048 Hz using a custom-written data acquisition program (Lab VIEW, National Instruments Corp., Austin, TX). Before sampling, the EMG signals were analogue band-pass filtered (high-pass 10 Hz, low-pass 1000 Hz) to remove unwanted noise and possible movement artefacts in the low-frequency region and to eliminate aliasing and other artefacts in the high-frequency region. The EMG data were recorded between the onset and the end of each 6-s sprint. EMG recording was by a digital trigger coincident with the start of the 6-s sprint and data collection stopped by a digital signal at the end of the sprint. After additional high-pass filtering (at 20 Hz) to eliminate movement artefact, root mean square (RMS) was calculated from each sprint. Also for each sprint, we calculated the neuromuscular efficiency (NME) as the ratio of the TW done divided by RMS activity of the VL (NME = TW/RMS). This ratio was considered to represent an indicator of the peripheral muscle contractility [17], [28].

### Muscle Sampling and Analysis

On the day of the 10×6-s cycle test, one incision was made under local anesthesia (5 mL, 1% Xylocaine) into the vastus lateralis muscle of each subject. The incision was used for the pre-exercise biopsy, closed with steri strips and subsequently used for the post-exercise biopsies. Pre- and post-exercise biopsy samples were taken with the needle inserted at different angles, with manual suction applied for all samples. The first muscle sample was taken at rest (before warm-up) while the subject was lying on a bed. The second muscle sample was obtained immediately following the cessation of the 10×6-s test, while the subjects remained on the cycle ergometer. The time from the end of the last sprint to placing the muscle sample in liquid nitrogen was <20 s. A further biopsy was taken after 6 min of exercise completion while subjects were resting on a couch. The samples were immediately removed from the biopsy needle and stored at −80°C until subsequent analysis. Freeze-dried muscle was dissected free from visible blood, fat, and connective tissue. Due to insufficient muscle sample size for one subject, muscle biopsy data was reduced to 7 subjects.

### Muscle [H^+^]

Freeze-dried muscle samples (1.8–2.3 mg) were homogenized on ice for 2 min in a solution containing sodium fluoride (NaF) (10 mM) at a dilution of 30 mg of dry muscle per milliliter of homogenising solution. The muscle homogenate was then placed in a circulating water bath at 37°C for 5 min before and during the measurement of pH. The pH measurement was made with a microelectrode (MI-415, Microelectrodes Inc, Bedford, NH) connected to a pH meter (SA 520, Orion Research Inc, Cambridge, MA).

### Muscle Metabolites

Freeze-dried rest and post-exercise muscle samples (2–3 mg) were enzymatically assayed for ATP, PCr and lactate (MLa^-^) concentration. ATP, PCr and MLa^-^ were extracted from muscle samples by the addition of 6% perchloric acid, before being centrifuged (10,000 g×10 min). The supernatant was removed and neutralized by the addition of 2.4 mol/L KOH and 3 mol/L KCl. Samples were centrifuged again and the supernatant was stored at −80°C. ATP, PCr and MLa^−^ concentration were measured using a previously described method [29].

### Statistical Analysis

Data are presented as mean ± SD within the text, and as mean ± SEM in the figures. The distribution of each variable was examined with the Kolmogorov Smirnov normality test. Sphericity was verified with a Mauchly's test. A one-way (i.e., sprint number) ANOVA with repeated measures was used to locate the significant differences in each dependent variable over time. Where a significant effect was detected, differences were located with *post hoc* paired *t*-tests with Bonferroni correction. For each variable, the percentage changes with 90% confident limits (CL) were also calculated to determine the magnitude of the effect. Individual relationships between variables were studied by means of regressions analysis and curve fitting using SPSS (version 13.0, Chicago, IL). Significance was accepted *a priori* at *P*<0.05.

## Results

### Changes in Mechanical Data

**Figure 2:**
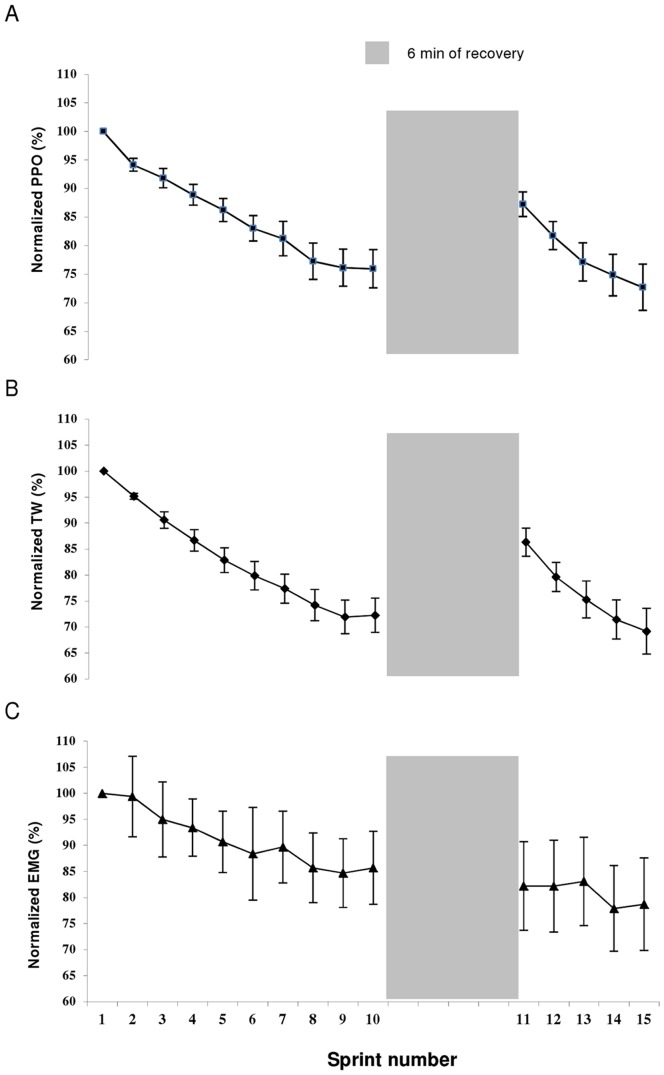
Average peak power output (A), total work done (B) and EMG amplitude (root mean square) (C) of the vastus lateralis muscle for the entire group over the 15 sprints. Data are presented as means ± SEM, expressed as a percent of sprint 1 (n = 8).

**Figure 3:**
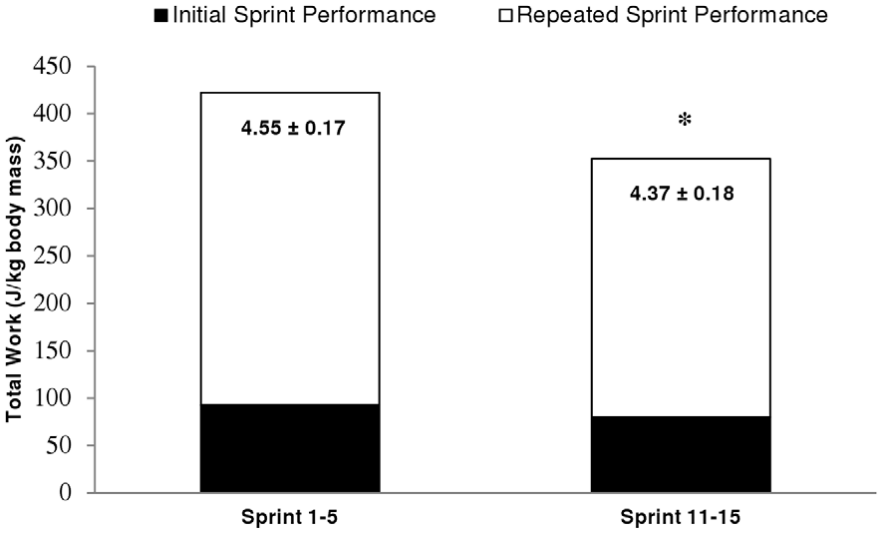
Total work done during five consecutive sprints (full bar height, TW_5SPT_) and the initial sprint (height of the dark lower portion, TW_1SPT_). The difference between these respective mechanical works is illustrated by the white upper portion of the bar. Numeric values (mean ± SEM) for the ratio of TW_5SPT_/TW_1SPT_ appear within the bars. Data for the bars are mean values and are presented as Joules per kilogram body mass (n = 8). * Significant differences between the ratios, P<0.05.

The PPO and TW values recorded during each of the 15, 6-s sprints are displayed in Figure 2. The decrease in PPO and TW after the first five sprints averaged 13.8±5.7% (90% CL: 10.0;17.6) and 17.1±6.1% (90% CL: 12.6;21.6) (P<0.001), respectively. The highest PPO and TW values were recorded during the first sprint and were significantly greater than the values attained in any of the other 14 sprints (P<0.05). Over the first ten sprints, average PPO and TW decreased by 24.1±9.4% (90% CL: 17.8;30.4) (P<0.001) and 27.2±9.4% (90% CL: 21.4;34.0) (P<0.001) from the maximal value (i.e., sprint 1), from 18.8±1.3 to 14.2±1.2 W·kg^−1^ and 92.8±6.3 to 66.7±6.7 J·kg^−1^, respectively. During sprint 11, following 6 min of passive rest, PPO (16.4±0.8 W·kg^−1^) and TW (79.8±4.8 J·kg^−1^) values recovered significantly in relation to those achieved in sprint 10 (P<0.001), but remained 12.8±6.1% (90% CL: 8.7;16.9) and 13.7±7.7% (90% CL: 8.5;18.9) (P<0.005) lower, respectively, than the values achieved during the first sprint. Over the last five sprints (11 to 15), following the recovery period, PPO and TW decreased by 17.0±8.7% (90% CL: 11.2;22.8) (P<0.01) and 20.3±9.4% (90% CL: 14.0;26.6) (P<0.001), respectively. During the last sprint (sprint 15), PPO (13.6±1.6 W·kg^−1^) and TW (63.7±8.8 J·kg^−1^) were 27.3±11.5% (90% CL: 19.6;35.0) and 30.8±12.4% (90% CL: 22.5;39.1) lower (P<0.001), respectively, than the maximal value obtained during the first sprint. The TW_5SPT_/TW_1SPT_ ratio between sprint 11 to 15 was significantly lower than the values obtained from sprint 1 to 5 (3.9±2.6%; (90% CL: 2.2;5.6); P<0.01; Figure 3).

### Changes in Muscle Metabolites and pH

Table 1 shows mean muscle metabolite concentrations and pH values before, after the first ten sprints, and following 6 min of recovery. There was a 29.5±9.6% (90% CL: 22.4;36.6) decrease in ATP content after sprint 10 (P<0.001). Following the 6 min recovery period, ATP was resynthesized to 92.6±6.0% (90% CL: 88.2;97.0) of the resting value, and was not significantly different from the resting value (P = 0.19). The PCr content of the muscle after sprint 10 was 46.9±21.4% (90% CL: 31.2;62.6) of the resting value (P<0.001). PCr was resynthesized to 85.3±10.3% (90% CL: 77.7;92.9) of the resting value by 6 min into the recovery, which remained significantly lower than the resting value (P<0.01). MLa^-^ content increased from rest by 921±232% (90% CL: 751;1091) (P<0.001) after sprint 10 and then decreased significantly to 559±184% (90% CL: 424;694) (P<0.001) following the 6 min recovery period. Muscle H^+^ concentration increased (P<0.001) to 191±50% (90% CL: 154;228) after sprint 10. Following the 6-min recovery period, H^+^ accumulation decreased to attain 144±32% (90% CL: 121;168) of the resting value (P<0.001). Muscle pH decreased significantly (P<0.001) by 0.27±0.12 pH (90% CL: 0.18–0.36) units following the first 10 sprints and increased slightly (0.12±0.06 units; 90% CL: 0.08–0.16) during the 6 min recovery. Thus, pH remained depressed in comparison with resting values (P<0.01) after the 6-min passive recovery period.

**Table 1:** Muscle ATP, PCr, Lac, H^+^ and pH in the vastus lateralis muscle at rest (Pre), following the completion of ten, 6-s cycling sprints interspersed with 30 s of recovery (Post 0), and after 6 min of recovery (Post 6).

Variable	Pre	Post 0	Post 6
ATP, mmol/kg dm	20.2±2.8	14.4±3.2 *	19.1±3.4 †
PCr, mmol/kg dm	76.2±9.8	37.0±21.4 *	63.3±17.8 * †
Lac, mmol/kg dm	12.2±4.5	108.4±34.0 *	64.1±19.7 * †
H^+^ (mmol/L)	106.8±16.0	198.4±34.0 *	151.0±24.3 * †
pH	6.95±0.07	6.75±0.13 *	6.84±0.12 * †

Values are means ± SD, *N* = 7 men. ATP = adenosine triphosphate; PCr = phosphocreatine; Lac = lactate; H^+^ = hydrogen ions; dm, dry muscle. * Significantly differences from Pre values, † Significant differences from Post 0 values, P<0.05.

### Changes in EMG Activity

The temporal profiles of the EMG amplitude (RMS) across the repeated sprints are shown in Figure 2 C. When compared with the first sprint, the decrease in RMS after the first five sprints averaged 9.2±6.2% (90% CL: 5.0;13.4) (P<0.01). The value for RMS, which was highest during the first sprint, decreased over the first ten sprints by 14.3±7.6% (90% CL: 9.2;19.4) (P<0.01), from 0.484±0.03 to 0.415±0.03 mV. After the 6 min of passive rest between sprint 10 and sprint 11, RMS during sprint 11 (0.398±0.03 mV) did not show any recovery from sprint 10, and was 18.3±12.4% (90% CL: 10.0;26.6) lower than the initial values recorded during the first sprint (P<0.001). During subsequent sprints (11 to 15), there was a further significant decrease (P<0.05) in RMS. Values for sprint 15 were 4.4±5.4% (90% CL: 0.8;8.0) (P<0.05) and 22.1±11.0% (90% CL 14.7;29.5) (P<0.001) lower than the values achieved in sprint 11 and sprint 1, respectively.

**Figure 4:**
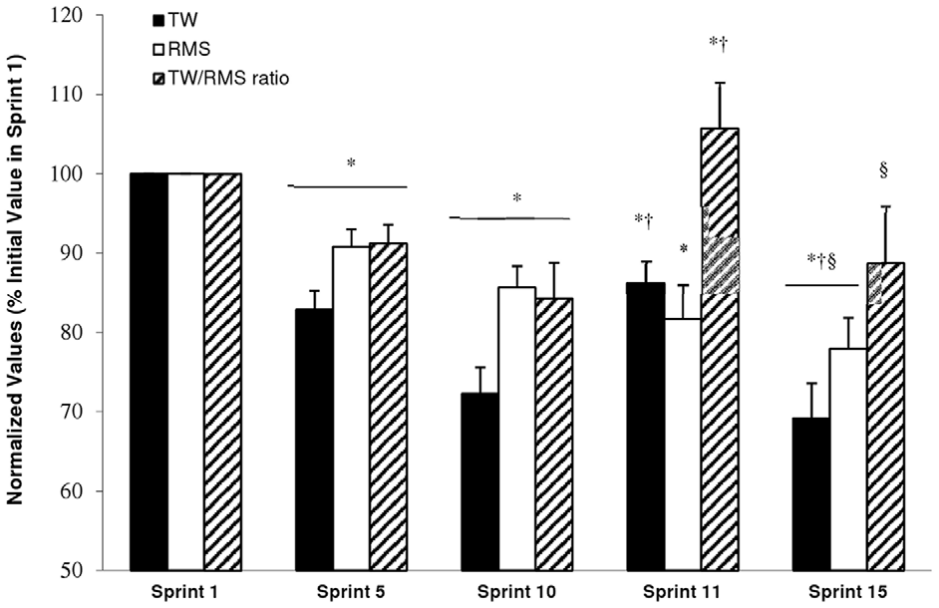
Evolution of the total work done (TW, black column), EMG root mean square of the vastus lateralis muscle (RMS, white column), and TW/RMS ratio (cross-hatch column) for the entire group over selected sprints. Data are presented as means ± SEM, expressed as a % of sprint 1 (n = 8). * Significantly different from Sprint 1, P<0.05; † Significantly different from Sprint 10, P<0.05; § Significantly different from Sprint 10, P<0.05.

Figure 4 shows for all subjects the normalized TW/RMS ratio (i.e., NME) for the VL muscle during the 15×6-s cycling test. The TW/RMS ratio, expressed as a percentage of the first sprint value, decreased in sprint 5 (−8.6±7.1%; 90% CL: −3.8;−13.4); P<0.01) and in sprint 10 (−15.1±13.6%; 90% CL: −6.0; −24.2); P<0.01) with respect to the initial value in sprint 1. The 6-min recovery period induced an increase in the TW/RMS ratio in sprint 11, which was significantly greater (P<0.001) than that found in sprint 10 (+20.7±8.5%; 90% CL: 15.0;26.4) but not significantly different in comparison with sprint 1 (P = 0.27). Values for sprint 15 were 16.8±7.6% (90% CL: 11.7;21.9) (P<0.001) lower than values achieved in sprint 11. Thus, NME was significantly affected by the intermittent exercise and the subsequent recovery period.

### Relationship between Variables

**Figure 5:**
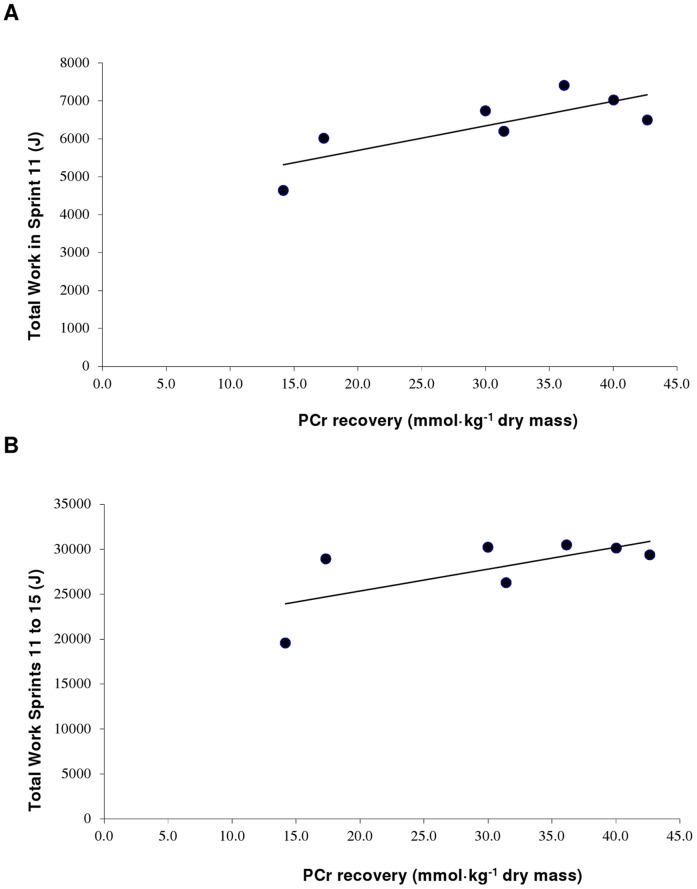
Muscle phosphocreatine (PCr) recovery following the 6 min of passive recovery was positively related to: A) the total work done during sprint 11 (r = 0.79, P<0.05) and, B) the total work done across the five consecutive sprints (r = 0.67, P<0.05).

PCr resynthesis (mmol·kg^−1^ dw) after the 6 min of rest was positively correlated with both TW completed in both sprint 11 (r = 0.79, P<0.05; Figure 5A) and during the last five sprints (i.e., sprints 11 to 15) (r = 0.67, P<0.05; Figure 5 B). No further statistical correlations were found between any of the performance variables in the second bout of five sprints and changes in any of the muscle metabolites. There were also no significant correlations between the recovery of sprint performance and change in EMG.

## Discussion

This, to our knowledge, is the first study to characterize the relationship between the recovery of repeated-sprint performance and some of its key putative physiological determinants. The main findings of this study were as follows: 1) there were significant decreases in both muscle metabolites and net motor unit activity during the prior repeated-sprint exercise; 2) the partial restoration of repeated-sprint performance was correlated with the resynthesis of PCr, but not with the removal of H^+^; net motor unit activity remained low during the recovery from the initial 10 sprints; 3) repeated-sprint exercise performance recovered to a lesser extent than single-sprint performance; 4) the lower mechanical power maintenance during subsequent repeated-sprint exercise, following the recovery period, was more likely limited by intramuscular factors than by neural adjustments (as inferred by the ∼2-fold greater decrease in the TW/RMS ratio).

### PCr Content and RSE

While a correlation between PCr resynthesis and the recovery of single-sprint performance has previously been reported [12], [13], we report for the first time that there is a significant correlation between the resynthesis of PCr and the recovery of repeated-sprint performance (r = 0.67; Fig. 5B). This finding contrasts with the results of a previous study assessing the recovery of single-sprint performance (i.e., two consecutive 30-s efforts separated by 4 min of recovery), where PCr resynthesis was only correlated with the recovery of power output during the initial 10 s, but not during the last 20 s of sprint two [12]. In that study muscle PCr stores were almost completely depleted during the first ten seconds of the second sprint, and the contribution of PCr during the last 20 s was minimal [12]. The 30-s recovery intervals between consecutive sprints in the present study have been reported to be sufficient to allow significant PCr resynthesis and, therefore, considerable contribution from PCr to ATP turnover, even during the latter sprints [3]. This probably explains why PCr resynthesis is correlated with the performance of 30 s of repeated all-out sprints (i.e., 5×6-s sprints; present study), but not with the performance of a 30-s continuous, all-out effort [12] and suggests that intramuscular PCr content is an important physiological determinant of performance during repeated-sprint exercise.

### H^+^ Accumulation

We also report for the first time that despite a persistent low muscle pH, power output partially recovered after 6 min of recovery (Fig. 2). Moreover, no significant correlations were found between the recovery of muscle pH and power output during single- or repeated-sprint exercise performance. Similarly, previous studies have shown that sprinting capabilities (i.e., power output) were restored faster than intracellular pH [13], [14]. Together with studies in isolated skinned and intact muscle fibres [30], [31], [32], [33], these results suggest that the removal of H^+^ is not causally related with force restoration following high-intensity exercise [12], [13], [34] and expand the notion that muscle acidosis might not be the main factor limiting the recovery of power output following sprint tasks. Thus, it seems that muscle energy supply (e.g., PCr resynthesis) is more important for the restoration of power output during repeated sprints than the recovery of muscle pH.

### Neuromuscular Adjustments

Net motor neuron activity (as evidenced by the EMG amplitude) decreased in parallel with changes in power output during the first ten sprints (Figure 2). Accordingly, the observed changes in EMG of the vastus lateralis, associated with the reductions in external mechanical output, permit the qualitative conclusion that changes in locomotor muscle power output were in similar direction to those of central neural locomotor output [24]. While caution should be used when inferring motor control strategies from EMG during dynamic muscle contractions [35], this implies that either the firing frequencies of some motor units were decreased and/or that there was a progressive reduction in the number of motor units recruited in the present study. The reduction in power output during the first ten sprints, however, makes it difficult to relate neural adjustments to performance as the lower EMG activity could also be the consequence, rather than the cause, of the reduced power output. Several lines of evidence which suggest that reductions in power output and neural adjustments may not be directly linked during repeated-sprint exercise are discussed below.

To our knowledge, the rate of recovery in neural processes following repeated-sprint exercise has not previously been considered. If neural adjustments make an important contribution to fatigue during repeated-sprint exercise, then we would expect to see the recovery of EMG amplitude to occur in parallel with the recovery of sprint power. In the present study, TW partially recovered (16%) at the start of the second set of repeated sprints (sprint 11), while EMG remained unaffected (compared with the values recorded in sprint 10). Similarly, Kalman and Cafarelli [36] reported a partial recovery of maximal voluntary contraction force, despite EMG amplitude remaining depressed, 5 min after an intermittent knee-extension contraction protocol performed to task failure. Furthermore, exercise-induced reductions in EMG amplitude have been reported in spite of the ability to maintain the same level of maximal and submaximal force [37], [38], [39], [40]. This supports our observation that the EMG amplitude can be dissociated from the recovery of TW during fatiguing repeated-sprint exercise.

Consistent with this dissociation between the recovery of TW and EMG following fatiguing exercise, there was an ∼2-fold greater decrease in the TW/RMS ratio in the second set of five sprints (sprint 11 to 15) than in the first five sprints (sprint 1 to 5), resulting from a greater and disproportionate decrease in mechanical power (i.e., TW) in relation to EMG. Thus, intramuscular mechanisms, most likely related to limitations in metabolic supply (e.g., PCr, see above), can explain most of recovery of TW and most of the subsequent TW decrements between sprints 11 to 15. Nevertheless, as all-out efforts require the maintenance of high motoneuron activity [41], [42], [43], the reduced motor unit activity may also contribute to the lower muscle power maintenance from sprints 11 to 15 [40]. Accordingly, the partial recovery of single-sprint performance (i.e., sprint 11) may be mainly attributed to the recovery of intramuscular processes subsequent to PCr resynthesis, despite suboptimal net motor unit activity, while the remaining decrement in TW may represent a persistent reduction in motor unit activity.

The observed reduction of EMG amplitude in sprint 11 and onwards (i.e., sprint 11 to 15), following the recovery period, may also be related to two additional factors that can enhance amplitude signal cancellation (i.e., a reduction in EMG amplitude signal that occurs when overlapping positive and negative phases of motor unit potentials cancel one another which is unrelated with changes in voluntary neural drive to the muscle) during fatiguing contractions [44], [45]. First, the slowing of motor unit firing rate resulting in a longer duration of motor unit action potentials have been observed during repeated maximal efforts [46], [47], and can lead to greater overlap between potentials and increase the amplitude cancellation [45]. However, it is also possible that the reported increase in action potential duration can augment EMG amplitude as the area of each motor unit potential increases [45]. Second, high-intensity dynamic muscle contractions, as in the present study, preferentially result in a selective fatigue of the type IIx fibres [48], which would decrease the amplitude and area of the largest motor unit potentials [49], resulting in a more narrow amplitude range for the motor unit potentials [45]. However, the recovery of muscle power output in parallel with intramuscular PCr content suggest an increased recruitment of the fast and powerful fast IIx fibers following the 6-min recovery period in the present study [48]. Therefore, while amplitude cancellation cannot be discarded, our results seem to suggest that a persistent suboptimal motor unit activity might have contributed to the inability of EMG amplitude to recover after the fatiguing maximal contractions performed in this study.

### Conclusions

The present study shows that locomotor skeletal muscle metabolism is severely imbalanced during repeated-sprint exercise in association with a reduced net motor unit activity. The recovery of locomotor muscle power output appears to follow a time course that is partially dependent on the restoration of intramuscular PCr metabolism, while muscle pH and net motor unit activity remains depressed. In addition, there was a disproportionate decrease in TW in relation to RMS in the second set of sprints (sprint 11 to 15), when compared with the first five sprints (sprint 1 to 5). Thus, we conclude that much of the inability to produce power output during repeated sprints is mediated by intramuscular fatigue factors probably related with the control of high-energy phosphate metabolism (i.e., ATP and PCr).
